# Dimethyl Fumarate ameliorates pulmonary arterial hypertension and lung fibrosis by targeting multiple pathways

**DOI:** 10.1038/srep41605

**Published:** 2017-02-02

**Authors:** Agnieszka P. Grzegorzewska, Francesca Seta, Rong Han, Caitlin A. Czajka, Katsunari Makino, Lukasz Stawski, Jeffrey S. Isenberg, Jeffrey L. Browning, Maria Trojanowska

**Affiliations:** 1 Boston University School of Medicine, Arthritis Center/Rheumatology, Boston, MA, USA; 2 Boston University School of Medicine, Vascular Biology Section, Boston, MA, USA; 3 Heart, Lung, Blood and Vascular Medicine Institute, University of Pittsburgh, USA; 4 Division of Pulmonary, Allergy and Critical Care Medicine, University of Pittsburgh School of Medicine, USA; 5 Boston University School of Medicine, Microbiology, Boston, MA, USA.

## Abstract

Pulmonary arterial hypertension (PAH) is a fatal condition for which there is no cure. Dimethyl Fumarate (DMF) is an FDA approved anti-oxidative and anti-inflammatory agent with a favorable safety record. The goal of this study was to assess the effectiveness of DMF as a therapy for PAH using patient-derived cells and murine models. We show that DMF treatment is effective in reversing hemodynamic changes, reducing inflammation, oxidative damage, and fibrosis in the experimental models of PAH and lung fibrosis. Our findings indicate that effects of DMF are facilitated by inhibiting pro-inflammatory NFκB, STAT3 and cJUN signaling, as well as βTRCP-dependent degradation of the pro-fibrogenic mediators Sp1, TAZ and β-catenin. These results provide a novel insight into the mechanism of its action. Collectively, preclinical results demonstrate beneficial effects of DMF on key molecular pathways contributing to PAH, and support its testing in PAH treatment in patients.

Pulmonary arterial hypertension (PAH) is a life-threatening disease characterized by vasoconstriction and vascular remodeling of pre-capillary pulmonary arterioles, leading to vascular rarefaction and remodeling, restriction of blood flow, increases in pulmonary arterial pressure (PAP) and pulmonary vascular resistance, and eventually right ventricular failure and death^1^. One of the known PAH risk factors is a pre-existing connective tissue disease, e.g. systemic sclerosis (SSc). SSc is characterized by autoimmunity, vasculopathy and fibrosis causing damage in multiple organ systems. Patients with SSc and PAH (SSc-PAH) have a poorer response to treatment and a worse prognosis (greater mortality) than other subgroups of PAH. Systemic vasculopathy and chronic inflammation are likely factors contributing to the more severe PAH disease manifestation in those patients^2^.

The pathological processes underpinning vascular remodeling in PAH are complex and involve interactions between different cell types within the vascular wall with further contributions from the immune and circulating progenitor cells. In response to injury, pulmonary endothelial cells (ECs) undergo phenotypic alterations resulting in secretion of excessive amounts of numerous mediators, including growth factors mitogenic for smooth muscle cells (SMCs) and fibroblasts, as well as chemokines that attract inflammatory cells. Due to the altered paracrine signaling proliferation of SMCs in PAH is enhanced, while apoptosis is depressed resulting in muscularization of peripheral small pulmonary arteries (PAs) and increased vasoconstriction^3^,^4^. It has also been recognized that adventitial fibroblasts play an important role in PAH not only as principal contributors to the pathological extracellular matrix (ECM) remodeling resulting in fibrosis and increased vascular stiffness, but also as mediators of vascular inflammation through the recruitment and activation of macrophages^5^.

The role of reactive oxygen species (ROS)-driven oxidative stress in the pathological process of vasculopathy and hypertension is well established. Excessive ROS diminishes nitric oxide (NO) bioavailability leading to increased vasoconstriction and damages the vasculature by inducing inflammation and fibrosis^6^. Altered redox homeostasis is consistently observed in PAH patients and there is growing evidence for ROS contributing to PAH development and progression^7^. The oxidative stress response is a central system designed to counteract the damaging effects of redox imbalance. This response includes expression of a broad gene panel encompassing protective anti-oxidant enzymes, enzymes to reduce reactive oxygen species and detoxifying agents (Phase II enzymes)^6^. The transcriptional regulator nuclear factor (erythroid-derived 2)-related factor 2 (NRF2) is a key regulator of the antioxidant genes^8^. Importantly, NRF2 function is linked to NFκB signaling with activation leading to an anti-inflammatory response^9^. The importance of this effect is best illustrated by the excessive inflammatory response to injury observed in Nrf2−/− mice stemming from increased NFκB activation, and increased TNFα, IL-1, IL-6 and ICAM expression^10^,^11^.

A wide range of NRF2 pathway activating agents, including dimethyl fumarate (DMF), have been tested in variety of disorders^12^. DMF elicits its potent anti-inflammatory response partly by directly targeting the NFκB signaling pathway through a covalent modification of p65^13^. In contrast to the anti-oxidative and anti-inflammatory effects of DMF, which have been widely studied, the anti-fibrotic effects of DMF remain largely unexplored. Fumarates (DMF/MMF) have been successfully used to treat two autoimmune and inflammatory diseases, psoriasis and multiple sclerosis (MS), with a favorable safety profile^14^,^15^.

Current strategies for the treatment of PAH include targeting deficiencies in NO signaling (PDE-type 5 inhibition), enhancing nucleotide signaling via cAMP PGI2 (prostacyclin analogs), or limiting excessive ET-1 effects (ET receptor blocker). Although effective in reducing vasoconstriction, pharmacologic treatment of PAH continues to have significant limitations^16^. The goal of this study was to assess the effectiveness of DMF as a therapeutic agent for PAH using patient-derived pulmonary cells and murine models of PAH. To address the important component of SSc-PAH, which is fibrosis, we tested DMF in a bleomycin mouse model. Our results demonstrate that DMF treatment is effective in reducing inflammation, oxidative damage and fibrosis in the *in vivo* experimental models and *in vitro* patient-derived cells, therefore supporting the testing of DMF/Tecfidera^®^ in PAH.

## Results

### DMF ameliorates pathological hemodynamics in chronically hypoxic mice

**Figure 1:**
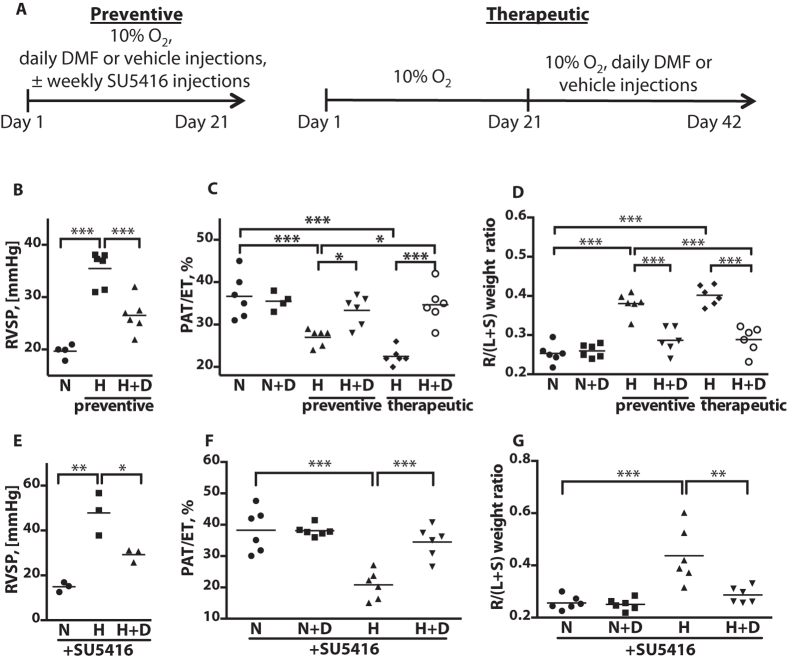
DMF abrogates hypoxia- and hypoxia/SU5416- induced increase in RV blood pressure and RV hypertrophy *in vivo.* **(A)** Experimental design for preventive and therapeutic DMF treatment modes. In the preventive mode mice were kept in normoxia (N) or hypoxia (H) (10% O_2_) for 21 days and injected daily with 90 mg/kg DMF (D) or vehicle. In the therapeutic mode mice were kept in hypoxia for a total of 42 days with daily DMF injections starting at day 21. In the hypoxia/SU5416 model, mice were kept in hypoxia for 21 days and injected weekly with 20 mg/kg SU5416. **(B,E)** The RV systolic blood pressure (RVSP) was measured by catheterization for the mice in the preventive experiment and hypoxia/SU5416 mice. n = 6 mice per group. **(C,F)** Pulmonary acceleration time (PAT) and PAT as a fraction of ejection time (PAT/ET) were measured. n = 4–6 mice per group. **(D,G)** The weight ratios of the right ventricle to the left ventricle plus septum (S) (RV/(LV + S)) were calculated as indices of RV hypertrophy. n = 6 mice per group. *P < 0.05, **P < 0.01, ***P < 0.001.

To assess the efficacy of DMF to treat primary outcomes of PAH including increased RV pressure and RV hypertrophy we employed chronic hypoxia and hypoxia/SU5416 mouse models. In the initial experiment, mice were challenged with hypoxia (10% O_2_) or normoxia for 21 days (Fig. 1A, Preventive). Experimental hypoxic and normoxic mice received daily ip injections of DMF (90 mg/kg), while control hypoxic and normoxic mice were injected with vehicle. Direct measurement of right ventricular systolic pressure (RVSP) via closed chest catheterization through the right jugular vein showed increased RVSP in hypoxic mice, which was significantly lower in animals treated with DMF (Fig. 1B). Doppler echocardiographic imaging of the pulmonary artery blood flow is an accurate, fast and non-invasive method of assessing the changes in PAP. Shortened pulmonary acceleration time (PAT) and decreased PAT/ET(ejection time) correlate with increased RVSP^17^. The echocardiographic measurement confirmed the catheterization results (Fig. 1C). In addition, assessment of RV hypertrophy, calculated as the weight of the right ventricle divided by the weight of the left ventricle and septum, revealed that DMF treatment prevented right heart hypertrophy (Fig. 1D). Thus, this initial experiment demonstrated that DMF is capable of preventing increased RVSP and RV hypertrophy in hypoxic mice. To test whether DMF treatment could reverse pre-existing pathological changes, hypoxia challenge was extended to 42 days, and DMF or vehicle was administered daily starting from day 21 (Fig. 1A, Therapeutic). Mice exposed to prolonged hypoxia showed further worsening of PAT/ET and RV hypertrophy and DMF treatment significantly normalized PAT/ET and RV hypertrophy as compared to mice challenged with hypoxia for 21 days, as well as 42 days (Fig. 1C,D). To test DMF in more severe PAH model we employed hypoxia/SU5416 mice, where in addition to 3 weeks of hypoxia mice were injected weekly with 20 mg/kg SU5416 (Fig. 1A)^18^. Hypoxia/SU5416-treated mice developed higher RVSP (~50 mmHg for hypoxia/SU5416 as compared to ~35 mmHg for hypoxia alone), worse PAT/ET (~20 for hypoxia/SU5416 as compared to ~25 for hypoxia alone) and more pronounced right heart hypertrophy (~0.45 for hypoxia/SU5416 as compared to ~0.4 for hypoxia alone) (Fig.1B–G). DMF significantly inhibited hypoxia/SU5416- induced hemodynamic changes (Fig.1B–G). These results indicate that DMF effectively prevents and reverses elevated RVSP and RV hypertrophy in murine PAH models.

### DMF mitigates oxidative stress damage and inflammation in lung

**Figure 2:**
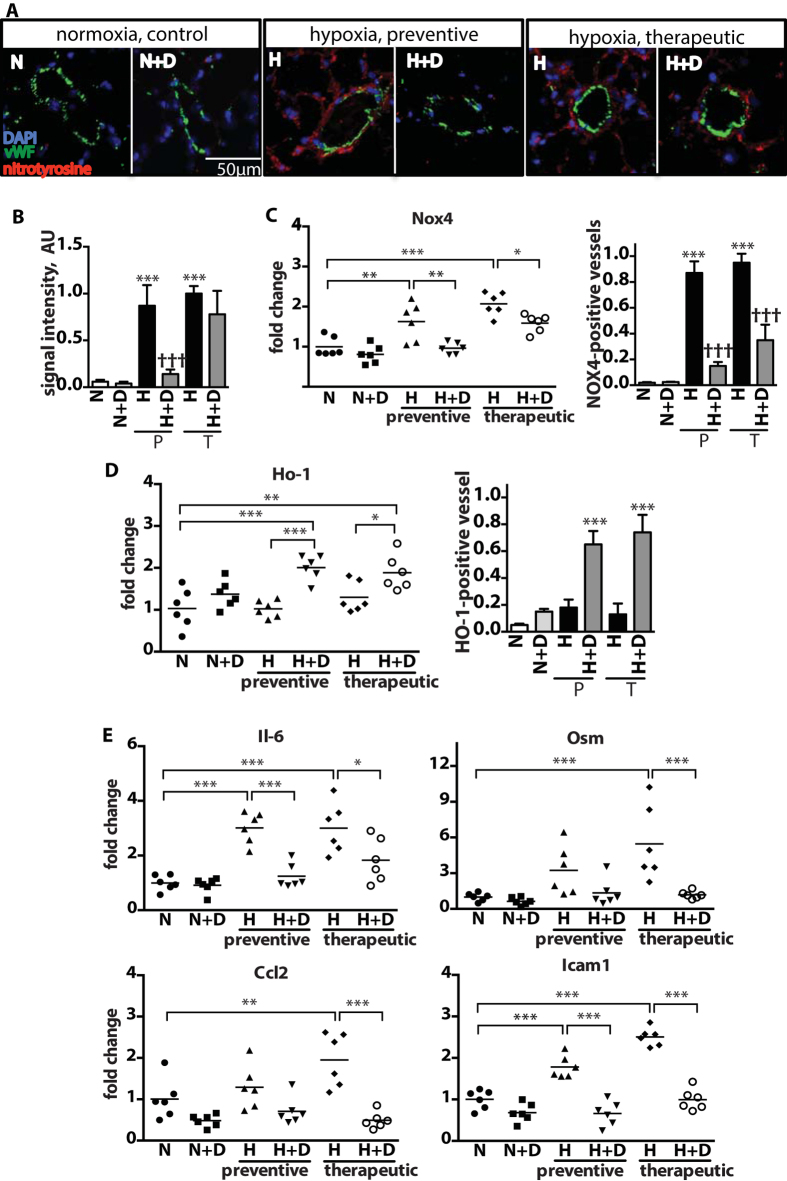
Attenuation of hypoxia-induced pulmonary oxidative stress damage and inflammation by DMF treatment. (**A**) Representative pictures of immunofluorescent staining of mouse lungs using anti-vWF antibody and anti-nitrotyrosine antibody as a marker of oxidative damage. **(B)** Quantification of the mean fluorescence intensity of nitrotyrosine staining from 5 fields of view per mouse. n = 4 mice per group. normoxia (N), hypoxia (H), DMF treatment (D), preventive treatment (P), therapeutic treatment (T). **(C,D,E)** qPCR quantification of relative mRNA levels in a whole lung. **(C)** Gene expression and quantification of vessels immunoreactive for NOX4 and **(D)** HO-1. Vessels were counted from 5 fields of view per mouse. n = 4 mice per group. *P versus normoxia (N); ^†^P versus hypoxia (H). **(E)** Gene expression of Il-6 family cytokines: Il-6 and Osm, Ccl2, and Icam1. n = 6 mice per group. Data shown as mean ± SD. *P < 0.05, **P < 0.01, ***P < 0.001.

We next assessed the ability of DMF to alleviate oxidative stress in hypoxic lungs. The extent of damage caused by oxidative stress was visualized by nitrotyrosine immunofluorescent staining and was shown to be prominently upregulated by hypoxia (Fig. 2A,B). DMF significantly reduced nitrotyrosine formation in the preventive treatment group, but had insignificant effect in the therapeutic group. In the therapeutic setting, reversal of nitrotyrosine formation would require substantial protein turnover which was not expected within this three-week treatment window. Likewise, the hypoxia-induced elevated gene and protein expression of Nox4 NADPH oxidase, a source of hydrogen peroxide in the pulmonary vasculature, was normalized by the preventive DMF treatment, but only partially by the therapeutic treatment (Fig. 2C). Interestingly, the NRF2 responsive anti-oxidative Ho-1 (heme oxygenase) gene and protein were significantly elevated by DMF in only hypoxic mice and not in normoxic mice (Fig. 2D). Both HO-1 and NOX4 proteins were localized in the perivasculature in hypoxic lungs and the representative micrographs of immunostainings are shown in Supplementary Fig. 1.

One of the pathological consequences of excessive production of oxidants is increased inflammation^6^. To determine the effect of DMF on lung inflammation, the expression of cytokines interleukin 6 (Il-6), oncostatin M (Osm), C-C motif chemokine ligand 2 (Ccl2) and the adhesion molecule Icam-1 in hypoxic and hypoxia/SU5416-treated mice was assessed using qPCR (Fig. 2E and Supplementary Fig. 2), while tissue infiltration by leukocytes and macrophages in preventive treatment group was quantified by immunostaining for cell type specific markers: CD45 and MAC3, respectively (Supplementary Fig. 3). DMF treatment effectively inhibited hypoxia-induced inflammation in all examined treatment groups (Fig. 2E, Supplementary Fig. 2,3). Together, these results indicate that DMF reduces pulmonary oxidative stress damage and inflammation *in vivo*.

### DMF inhibits STAT3-, NFκB- and cJUN-mediated activation of endothelial cells in vitro

**Figure 3:**
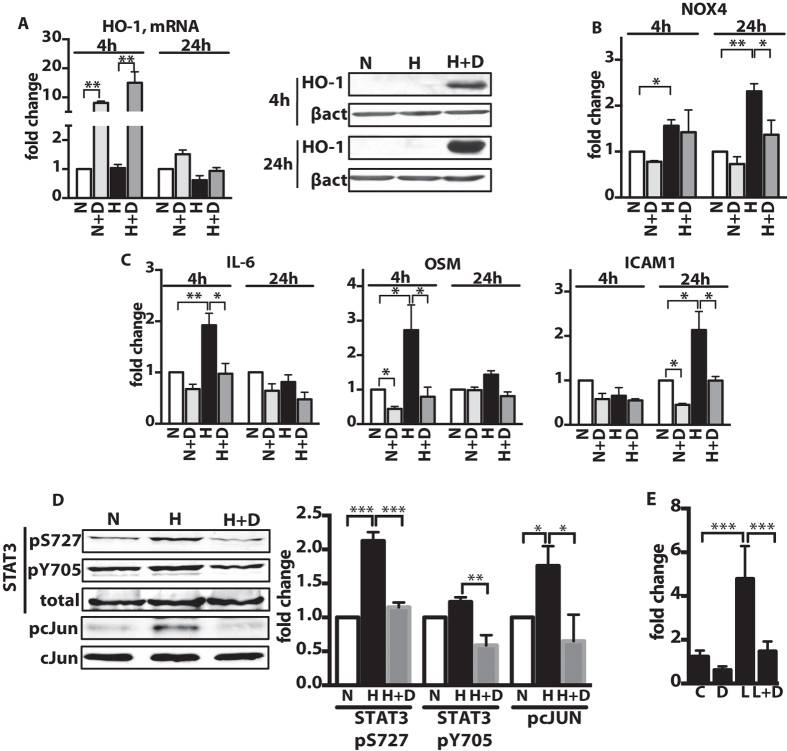
DMF inhibits pro-inflammatory gene expression in endothelial cells by suppressing NFκB, STAT3 and cJUN signaling. **(A,B,C,E)** HPAECs were incubated for up to 24 h in (H) hypoxia (2.5% O_2_) or (N) normoxia (21% O_2_) with 10 μM DMF (D) or DMSO. Relative gene expression was measured with qPCR: **(A)** HO-1**, (B)** Nox4, **(C)** pro-inflammatory genes: OSM, IL-6 and ICAM1. n = 4 independent experiments. **(D)** 10 μM DMF treatment reduced the phosphorylation of STAT3 and cJUN in HPAECs exposed to hypoxia for 4 h. Representative immunoblots and densitometry quantification are shown. n = 3 independent experiments. **(E)** NFκB activity luciferase reporter assay was performed in HPAECs in presence of 10 ng/ml LPS (L) and/or 30 μM DMF (**D**), control cells treated with DMSO (**C**). n = 3 independent experiments. Data shown as mean ± SD. *P < 0.05, **P < 0.01, ***P < 0.001. The uncropped images of selected immunoblots are shown in Supplementary Fig.14.

Injured endothelial cells are known to produce cytokines and chemokines that escalate the inflammatory activation of the vasculature^4^. The effect of DMF on activation of endothelial cells was investigated using human pulmonary artery endothelial cells (HPAECs) exposed to hypoxia (2.5% O_2_) in culture. Experiments to determine an optimal concentration of DMF *in vitro* found maximum HO-1 expression following treatment with 10 μM DMF (Fig. 3A). DMF (10 μM) significantly reduced basal mRNA levels of OSM and ICAM1 and prevented hypoxia-induced upregulation of NOX4, IL-6, OSM and ICAM1 (Fig. 3B,C), as well as the pro-fibrotic and pro-proliferative genes known to be involved in PAH pathology^3^: endothelin 1 (ET-1), connective tissue growth factor (CTGF), platelet derived growth factor beta polypeptide (PDGFB) and transforming growth factor TGFβ (Supplementary Fig. 3). We next examined the effects of DMF on the pro-inflammatory cell signaling pathways relevant to PAH^4^. Hypoxia induced phosphorylation of STAT3 (activating phosphorylation at residues S727 and Y705) and cJUN, while DMF treatment abrogated the hypoxia-induced phosphorylation of both proteins (Fig. 3D). Under hypoxic conditions, activation of NFκB was not consistently observed and, therefore, we examined lipopolysaccharide (LPS)-triggered NFκB activation in HPAECs. Based on previously published data in mouse splenocytes^19^, we used a higher dose of DMF (30 μM), which significantly diminished the activity of the NFκB luciferase reporter after LPS (10 ng/ml) stimulation (Fig. 3E). We next determined whether the anti-oxidative NRF2 pathway was required for the anti-inflammatory effect of DMF. Consistent with the previous study^19^, the knock down of NRF2 did not affect the ability of DMF to block the LPS-induced expression of pro-inflammatory genes in HPAECs (Supplementary Fig. 4). These observations show that DMF can prevent endothelial cell activation, possibly through DMF-mediated inhibition of major pro-inflammatory pathways: NFκB, STAT3 and cJun.

### Pulmonary vascular muscularization *in vivo* and hypoxia-induced changes in human pulmonary arterial vascular smooth muscle cells are reduced by DMF

**Figure 4:**
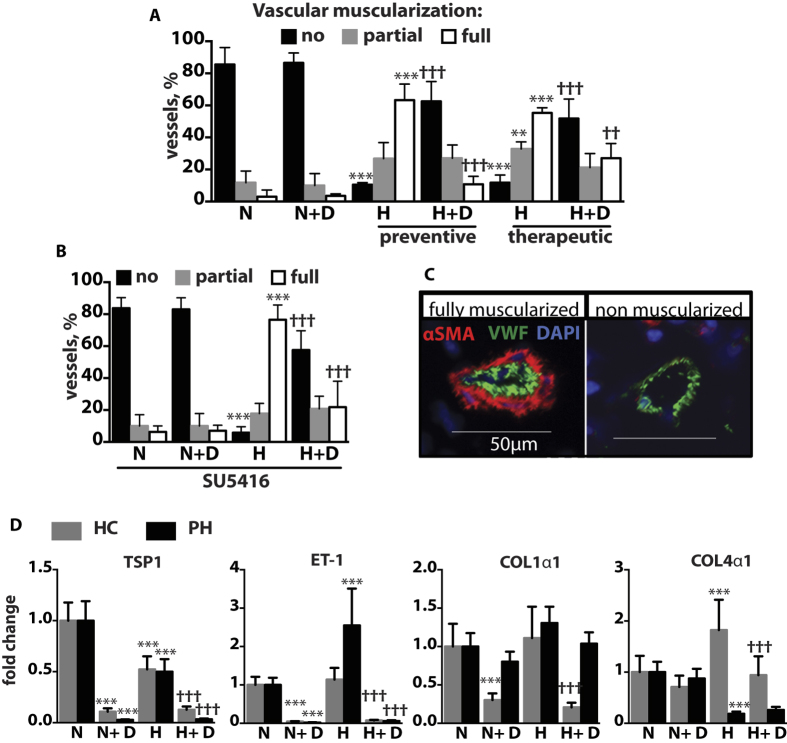
DMF inhibits vascular muscularization in hypoxic, hypoxic/SU5416 mice and suppresses hypoxia-induced gene expression in human PASMCs. **(A,B)** Intra-acinar vessels ranging from 20 to 70 μm in size were counted and categorized into non muscularized, partially muscularized, or fully muscularized. n = 4 mice per group. **(C)** Representative pictures of immunostaining of mouse lung sections with anti-vWF and anti-αSMA antibodies. **(D)** Smooth muscle cells were incubated in hypoxia (**H**) (1% O_2_) or normoxia (N) for 24 h with 50 μM DMF or DMSO. Relative gene expression was measured with qPCR. n = 1 donor for healthy control (HC) and n = 2 donors for pulmonary hypertension (PH) cell lines. Two independent experiments were performed, each in triplicate. *P versus normoxia(N); ^†^P versus hypoxia(H). Data shown as mean ± SD. *P < 0.05, **P < 0.01, ***P < 0.001.

To assess the effects of DMF on vascular remodeling, we performed immunostaining with antibodies to vWF, a vascular endothelium marker, and αSMA, a smooth muscle cell marker (Fig. 4C). Vascular muscularization was quantified and presented as the percent of peripheral pulmonary arterioles that were fully, partially or non-muscularized. The numbers of partially and fully muscularized vessels were elevated to a similar extent in mice challenged with hypoxia for 21 and 42 days and were higher in hypoxia/SU5416-treated mice. DMF significantly ameliorated vascular muscularization in both treatment groups in hypoxic mice as well as in hypoxia/SU5416-treated mice (Fig. 4A,B).

In our translational studies of direct DMF effect on vascular smooth muscle cells, we cultured pulmonary arterial vascular smooth muscle cells from the distal vasculature of lungs obtained from two patients with end-stage pulmonary hypertension and one healthy control. Cells were treated with normoxia or hypoxia (1% O_2_) for 24 h ± DMF (50 μM) and the PAH-related genes^20^ transcript levels were measured. We found that DMF treatment reduced angiostatic thrombospondin-1 (TSP1) transcript levels in healthy control (HC) and pulmonary hypertension (PH) HPASMCs under normoxic and hypoxic conditions (Fig. 4D). Acute hypoxia lowered TSP1 mRNA in HC and PH HPASMC. We previously reported that TSP1 mRNA levels increased after hypoxia (1% O_2_, 24 h) in HPASMC cultured under serum-restricted media conditions^21^. In contrast, we now find that hypoxia suppresses TSP1 mRNA in HPASMC cultured in complete medium. Since restriction of growth factors and serum in cultured SMC is known to limit cell cycle progression^22^, our new data might suggest that transcriptional regulation of TSP1 is cell cycle and growth factor linked in HPASMC.

Notably, ET-1 transcript levels were increased in hypoxic PH HPASMC following hypoxia challenge but not in HC cells, while DMF treatment resulted in decreased ET-1 mRNA levels in HC and PH HPASMC under both hypoxic and normoxic conditions (Fig. 4D). DMF treatment inhibited (COL)-1α1 and -4α1 transcript levels under normoxic conditions in HC cells. Likewise, DMF treatment inhibited hypoxia-induced changes in (COL)-1α1 and -4α1 mRNA in HC HPASMC but had no effect in PH HPASMC. In summary, DMF prevents and reverses vascular muscularization *in vivo* and reduces PAH-related gene expression in HPASMCs.

### Bleomycin-induced lung fibrosis is prevented by DMF treatment

**Figure 5:**
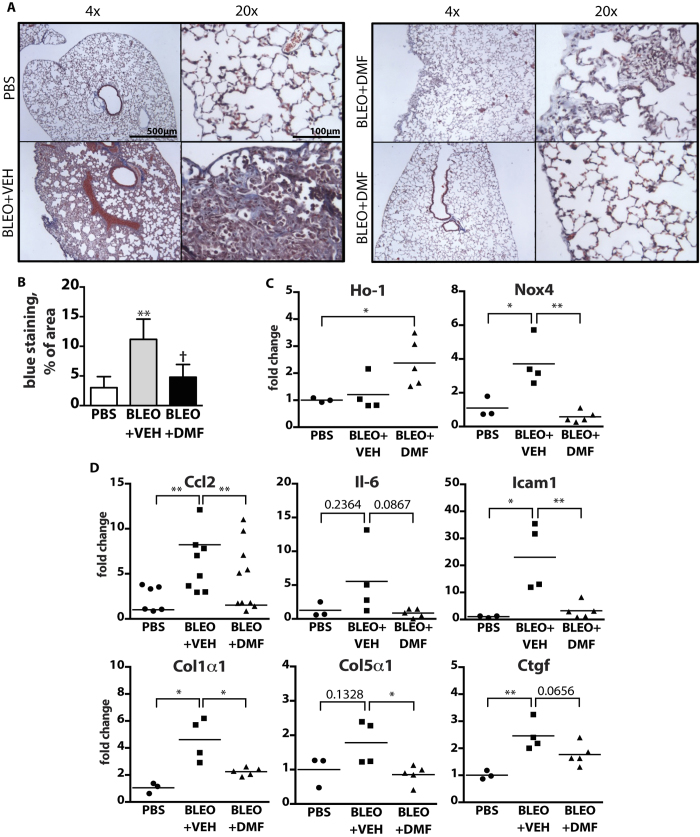
DMF prevents development of pulmonary fibrosis in bleomycin mouse model. Lung fibrosis was induced by bleomycin (BLEO) delivered through osmotic pump, PBS was used as a control. Mice were injected daily with 90 mg/kg DMF or vehicle (VEH) for 4 weeks. **(A)** Representative pictures of lung sections stained with Gomori trichrome for collagen (blue). n = 3–5 mice per group. **(B)** Quantification of blue area in Gomori trichrome staining expressed as percentage of the total tissue area. **(C,D)** Gene expression measured by qPCR in a whole lung tissue. *P < 0.05, **P < 0.01, ***P < 0.001.

The role of activated adventitial fibroblasts in pathogenesis of PAH has been recently appreciated^5^. In the course of this study, we noticed a significant reduction in the expression of pro-fibrotic mediators upon DMF treatment both *in vivo* and *in vitro* (Supplementary Fig. 5,6). Hypoxic and hypoxia/SU5416 mouse models are known for only moderate vascular remodeling and lack fibroproliferative changes that are characteristic to SSc-PAH. To address this question and directly investigate the anti-fibrotic potential of DMF, we utilized a pulmonary fibrosis model wherein fibrosis was induced by osmotic pump-delivery of bleomycin, and injected DMF daily for the entire duration of the experiment (4 weeks) (Fig. 5). Bleomycin-treated mice injected with vehicle developed pulmonary fibrosis as visualized by trichrome staining and its quantification of the lung sections (Fig. 5A,B, BLEO + VEH). In contrast, in three out of five mice treated with bleomycin plus DMF there were no detectable pathological changes in lung histology (Fig. 5A,B, BLEO + DMF, lower panel), while the other two DMF-treated mice exhibited some residual subpleural fibrotic lesions (Fig. 5A,B, BLEO + DMF, upper panel). In accordance to PAH mouse model observation, DMF upregulated expression of anti-oxidative Ho-1 and reduced bleomycin-induced expression of Nox4 (Fig. 5C). Consistent with the results of the histological analysis, DMF inhibited the expression of major pro-fibrotic genes that were upregulated by bleomycin treatment, including Col1α1, Col5α1, Ctgf, Et-1, and tissue inhibitor of metalloproteinase 1 (Timp1) (Fig. 5D). In addition, DMF also reduced the expression of the pro-inflammatory genes Ccl2, Il-6, and Icam-1 (Fig. 5D), which are known to be essential for the fibrotic processes in the bleomycin model. Taken together, DMF effectively prevented development of lung fibrosis *in vivo*.

### DMF blocks pro-fibrotic gene expression in human lung fibroblasts

The anti-fibrotic properties of DMF were examined further *in vitro* using TGFβ-stimulated human lung fibroblasts from healthy controls and SSc patients. A pilot experiment established that a higher concentration of DMF (90 μM) was needed to effectively block TGFβ (2.5 ng/ml) response without affecting cell viability (Supplementary Fig. 7). This result is consistent with a study in cultured human pulmonary and dermal fibroblasts, which utilized 100 μM DMF^23^,^24^.

**Figure 6:**
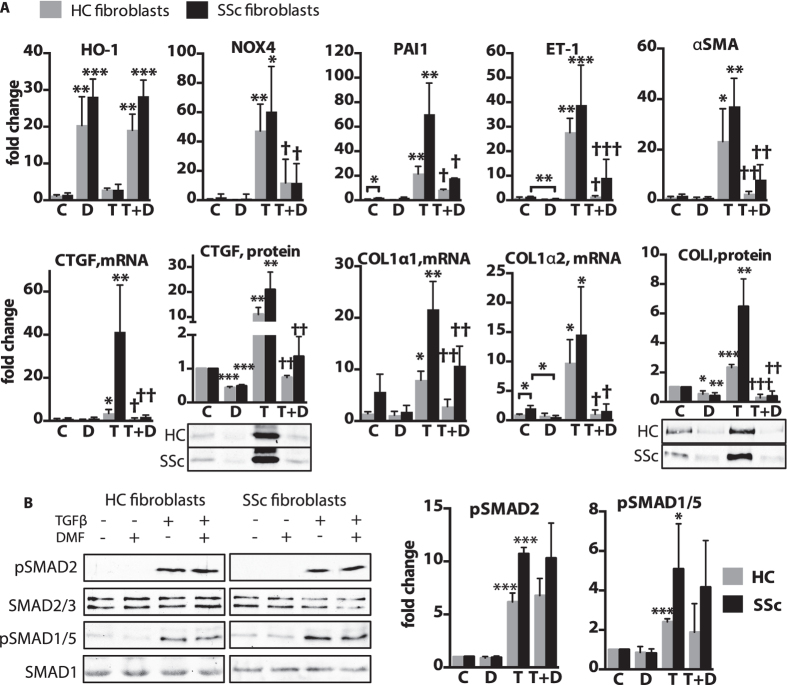
TGFβ –induced lung fibroblast activation is inhibited by DMF. (**A,B**) Human primary lung fibroblasts from healthy controls (HC) and scleroderma patients (SSc) were treated for (**A**) 24 h with 2.5 ng/ml TGFβ(T) and 90 μM DMF(D), control cells (**C**) were treated with vehicle (DMSO), or (**B**) pre-treated for 1 h with DMF(**D**) and then exposed to TGFβ(T) for 30 min. **(A)** Relative gene expression was measured with qPCR. CTGF and COLI proteins were measured by densitometry of immunoblots from the whole cell lysates. **(B)** Representative immunoblots and densitometry measurements of pSMAD2 and pSMAD1/5 levels. n = 3 cell lines per group. Data shown as mean ± SD. *P versus control (**C**); ^†^P versus TGFβ(T). *P < 0.05, **P < 0.01, ***P < 0.001. The uncropped images of selected immunoblots are shown in Supplementary Fig.14.

Lung fibroblasts obtained from SSc patients and healthy controls were stimulated with TGFβ for 24 h. As expected, TGFβ induced expression of the profibrotic genes, including COL1A1, COL1A2, CTGF, PAI1, ET-1, and αSMA in both SSc and healthy fibroblasts, with SSc fibroblasts showing a greater magnitude of response (Fig. 6A). DMF moderately reduced basal mRNA expression levels of ET-1, αSMA, COL1A1, COL1A2 (Supplementary Fig. 8), and significantly reduced basal protein levels of CTGF and collagen type I (Fig. 6A). The pro-fibrotic response to TGFβ in healthy and SSc fibroblasts was blocked by DMF in all the genes we examined (Fig. 6A). Knock down of NRF2 in fibroblasts demonstrated that the inhibitory effect of DMF on the TGFβ-mediated pro-fibrotic response was independent of NRF2 (Supplementary Fig. 9). In contrast NRF2 was required for DMF inhibition of TGFβ-induced expression of NOX4. Surprisingly, the major TGFβ-responsive pro-fibrotic signaling pathway involving SMAD2/3 and SMAD1/5 phosphorylation was not significantly affected by DMF treatment (Fig. 6B). The protein level of other known anti-fibrotic mediators PPARγ and FLI1 also did not change (Supplementary Fig. 10). Thus, DMF inhibits pro-fibrotic effect of TGFβ without affecting the SMAD pathway.

### DMF promotes βTRCP-dependent proteasomal degradation of β-catenin, TAZ and Sp1

**Figure 7:**
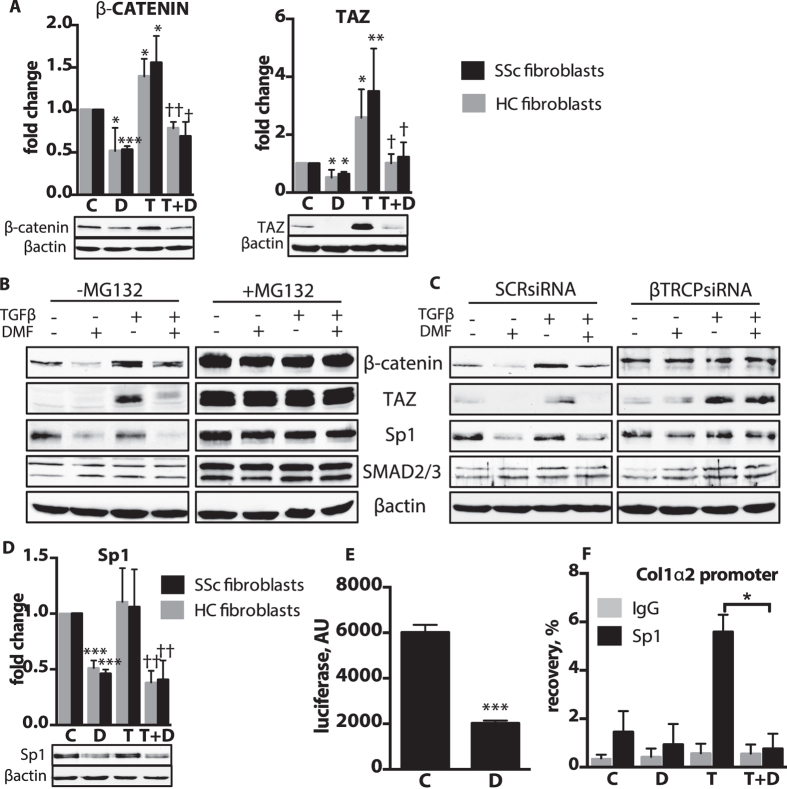
DMF promotes βTRCP-mediated degradation of Sp1, β-catenin and TAZ. (**A,D**) Lung fibroblasts from patients were treated for 24 h with TGFβ(T) and DMF(D) or vehicle (**C**). Representative immunoblots and densitometry measurements of β-catenin and TAZ. n = 3 cell lines per group. **(B)** Lung fibroblasts (IMR90) were pre-treated with proteasome inhibitor MG132 for 1 h and then exposed to TGFβ with DMF or vehicle for 24 h. **(C)** IMR90 were silenced with scrambled siRNA (SCRsiRNA) or βTRCP siRNA and then treated with TGFβ with or without DMF treatment for 24 h. Representative immunoblots are shown. (**D**) Representative immunoblots and densitometry measurements of Sp1. **(E)** Lung fibroblasts were transfected with COL1α2 promoter reporter and then treated for 24 h with DMF or vehicle. n = 3 independent experiments. **(F**) Chromatin immunoprecipitation using Sp1 antibody was performed after 24 h treatment with TGFβ and DMF or vehicle. The amount of recovered DNA was measured with qPCR. n = 3 independent experiments. Data shown as mean ± SD. *P versus control (**C**); ^†^P versus TGFβ(T). *P < 0.05, **P < 0.01, ***P < 0.001. The uncropped images of selected immunoblots are shown in Supplementary Fig. 14.

To elucidate further the anti-fibrotic mechanism of DMF, we examined other signaling pathways involved in the pathological fibrotic process. Because the Wnt/β-catenin pathway is implicated in pulmonary fibrosis^25^, we examined the effect of DMF treatment on the protein levels of the Wnt signaling effector molecule, β-catenin. Consistent with previous reports^26^, TGFβ upregulated the level of β-catenin protein and DMF significantly reduced both basal and TGFβ-induced levels of β-catenin in both healthy control and SSc fibroblasts (Fig. 7A). These findings were further corroborated in lung tissue sections from bleomycin treated mice where increased numbers of active (unphosphorylated) β-catenin-positive cells in the fibrotic regions were normalized by DMF treatment (Supplementary Fig. 11).

Degradation of β-catenin is coupled to degradation of the Hippo pathway effector, WW domain containing transcription regulator 1 (TAZ), which is also a major transactivator of the CTGF promoter^27^. Like β-catenin, protein levels of TAZ were elevated in response to TGFβ but reduced by DMF treatment (Fig. 7A). This observation suggested that DMF might regulate the protein stability of both β-catenin and TAZ. To explore this possibility, we treated fibroblasts with the proteasomal inhibitor MG132 before addition of TGFβ and DMF. Indeed, inhibition of the 26 S proteasome completely suppressed the ability of DMF to regulate protein levels of β-catenin and TAZ (Fig. 7B). Likewise, TGFβ upregulation of β-catenin and TAZ no longer occurred. It is possible that DMF could upregulate 26 S proteasome activity in a general fashion. However, we found that protein levels of SMAD2/3, another known 26 S proteasome target, were not affected by DMF treatment suggesting that the effects of DMF on proteasome activity were at least partially selective (Fig. 7B). We additionally verified that DMF does not alter the cytoplasmic-nuclear protein shuttling of TAZ/YAP (Supplementary Fig. 12).

Proteosomal degradation of β-catenin and TAZ is promoted by βTRCP, a component of the E3 ubiquitin ligase^27^. siRNA suppression of βTRCP (Supplementary Fig. 13), mitigated DMF-mediated degradation of β-catenin and TAZ (Fig. 7C). Interestingly, in the absence of βTRCP, TGFβ-mediated stabilization of TAZ was not affected, while β-catenin was no longer responsive to TGFβ treatment. These data suggest that although these two transcription co-factors experience common βTRCP-mediated proteasomal degradation, additional pathways specific for each factor contribute to their regulation. In addition, we verified that DMF stimulates βTRCP-specific degradation by showing that SMAD2/3 remained unchanged in the βTRCP silenced cells (Fig. 7C).

Since DMF reduced the levels of proteins regulated by βTRCP, we postulated that expression of the βTRCP target protein Sp1^28^, an essential regulator of collagen genes^29^, would be impacted. DMF reduced the basal Sp1 protein levels (Fig. 7D) and similar to the results for TAZ and β-catenin, proteasome inhibition blocked the effects of DMF. The effects of DMF on Sp1 levels were linked to βTRCP because knockdown of βTRCP eliminated the effect of DMF (Fig. 7B,C). To confirm the functional significance of Sp1 regulation by DMF, we employed chromatin immunoprecipitation and luciferase activity promoter assays. These assays revealed that COL1α2 promoter activity, as well as binding of Sp1 to the COL1α2 promoter, are significantly reduced by DMF (Fig. 7E,F).

In conclusion, the studies in human lung fibroblasts implicate βTRCP-dependent, 26 S proteasome-mediated degradation of Sp1, β-catenin and TAZ as a mechanism for the anti-fibrotic activity of DMF.

## Discussion

Despite recent improvements in PAH therapies, the mortality rate of PAH is approximately 55–65% at three years with a poorer response to treatment and a worse prognosis for SSc-PAH patients (an unadjusted risk of death of 2.9 when compared to idiopathic PAH)^2^,^30^. The complexity of PAH, including impaired redox homeostasis, abnormal vascular remodeling, adventitial fibrosis and immune cell activation suggests that simultaneous pharmacological modulation of multiple key pathways may be required for the effective treatment of PAH. Here we show that DMF has pleiotropic modes of action that may ameliorate PAH. DMF not only prevented the development of increased RVSP and RV hypertrophy in hypoxic and hypoxia/SU5416-treated mice, but also reversed pre-existing disease in a chronic hypoxia mouse model. DMF treatment efficiently reduced the contributions of pathogenic pathways in several cell types associated with PAH, including vascular and immune cells, as well as fibroblasts. The use of the chronic hypoxia and hypoxia/SU5416 mice as models of PAH imposes some limitations to this study. As described previously^31^, small rodent PAH models do not resemble all the features of human PAH and lack the autoimmune and fibrotic component of SSc. Therefore, we complemented our *in vivo* findings with extensive *in vitro* studies in human pulmonary vasculature cells.

The potent anti-inflammatory and anti-oxidative effects of DMF are of particular relevance as these pathways have been increasingly recognized as being pivotal in PAH pathogenesis. For example, IL-6 is elevated in serum and lung tissues of patients with PAH and Il-6 overexpressing mice spontaneously develop PAH^32^. Several studies have shown that increasing anti-oxidative potential and inhibiting key pro-inflammatory pathways such as NFκB is sufficient to prevent vascular remodeling in experimental models of PAH^33^,^34^,^35^. Our study demonstrates that DMF inhibits crucial pro-inflammatory pathways, including NFκB, STAT3 and cJUN in HPAECs, which correlates with decreased lung tissue infiltration by macrophages and immune cells *in vivo* and reduced expression of IL-6 in both hypoxic and bleomycin models. Others have shown that DMF inhibits HIF1α expression and function^36^, which might further explain the beneficial effects of DMF in PAH.

Pathological remodeling of pulmonary vessels characterized by muscularization of small arterioles as well as concentric intimal thickening and adventitial fibrosis is a central feature of PAH^3^,^37^. The ability of DMF to change the expression of key PH-related genes was tested using freshly harvested pulmonary arterial smooth muscle cells (PAMSC) from patients with and without PH. Treatment with DMF altered mRNA levels of several PAH-related genes including TSP1, ET-1. This is consistent with the recent findings indicating that in clinical PH TSP1, via CD47, activates ET-1 through suppression of cMyc^38^. Our results are limited by the small number of available human samples.

Recent studies strongly support the importance of activated adventitial fibroblasts as key contributors to vascular inflammation and fibrosis^37^. In addition to the excessive extracellular matrix deposition, activated adventitial fibroblasts have been shown to recruit and activate macrophages, and enhance inflammation via IL-6 production^5^. We show that in fibroblasts isolated from lungs of SSc patients or healthy controls, DMF diminished both basal and TGFβ-induced pro-fibrotic gene expression. In contrast to a previous report^39^, DMF did not affect canonical TGFβ signaling, as phosphorylation of SMAD2/3 and SMAD1/5 and protein levels of SMADs were not significantly altered by DMF. This discrepancy could be explained by the differences in cells used in the respective studies (rat kidney fibroblasts vs human lung fibroblasts). Interestingly, TGFβ-induced expression of pro-fibrotic and pro-inflammatory genes was efficiently blocked by DMF even in the absence of NRF2, whereas regulation of NOX4 expression was NRF2 dependent. This suggests that DMF blocks TGFβ transcriptional response through NOX4-independent pathways *in vitro*. However, reduction of NOX4 might contribute to the anti-fibrotic action of DMF *in vivo*, as NOX4 is linked to the development of pulmonary fibrosis^40^. Chronic hypoxic mice lack fibroproliferative changes, therefore the anti-fibrotic potential of DMF was further validated *in vivo* in a bleomycin-induced lung fibrosis model in which DMF efficiently prevented pathological collagen deposition and changes in pulmonary morphology, and significantly reduced expression of pro-inflammatory and pro-fibrotic genes.

Our exploration of the effects of DMF on TGFβ-induced fibrotic process in human lung fibroblasts has revealed a novel mechanism of action for DMF. DMF promotes the degradation of β-catenin and TAZ in human lung fibroblasts. β-catenin and YAP/TAZ are transcriptional effectors of the interconnected Wnt and Hippo pathways that play essential roles in tissue homeostasis by regulating genes determining cell fate, proliferation and apoptosis^41^. Both Wnt and Hippo pathways have been implicated in pulmonary fibrosis and PAH^25^,^42^,^43^, and are considered promising therapeutic targets for fibrotic disease and PAH^44^,^45^. Indeed, blocking Wnt signaling, downregulating β-catenin or YAP/TAZ prevented fibroblast activation *in vitro* and fibrosis development in animal models^42^,^46^. Here we show that DMF promotes proteasomal, βTRCP-dependent degradation of β-catenin and TAZ, while TGFβ mediates stabilization of those proteins, which may explain, at least in part, antagonistic effect of DMF on TGFβ signaling. Notably, in the βTRCP silencing experiments we observed different patterns of stabilization of β-catenin and TAZ by TGFβ, which might be due to the unknown effects of TGFβ and DMF on posttranslational regulators controlling stability of those proteins.

Although our study focused on regulation of ECM production, it is worth noting that β-catenin and TAZ might contribute to other features of PAH-associated fibroblasts and other vascular cells, including hyperproliferation and reduced apoptosis, and these pathways may also be affected by DMF treatment^25^,^47^. Finally, we show that DMF mediates proteasome-mediated degradation of a transcriptional activator, Sp1. Given the central role of Sp1 in basal and TGFβ-induced expression of ECM genes its degradation may represent an important mechanism of the anti-fibrotic effects of DMF^48^.

DMF/Tecfidera^®^ has been extensively used in the treatment of multiple sclerosis and while its NRF2 inducing properties are frequently highlighted, it is also recognized that DMF probably has multiple modes of action. In support of NRF2-based therapy in PAH, bardoxolone methyl (CDDO-Me), a NRF2 inducer^49^, has shown encouraging results in a Phase II study in PAH (http://reatapharma.com/reata-announces-initial-phase-2-pulmonary-arterial-hypertension-data-for-bardoxolone-methyl-and-planned-initiation-of-phase-3-study/) and anti-fibrotic effect in animal model^50^. DMF is especially attractive as an approved drug with a good safety record in both psoriasis and multiple sclerosis. In conclusion, this study strongly supports the testing of DMF as a therapy for patients with PAH.

## Materials and Methods

### Mice

6–8 week old male C57BL/6 mice were purchased from The Charles River. All experimental procedures were performed in accordance to protocol reviewed and approved by the Boston University Institutional Animal Care and Use Committee (protocol AN-15037). The investigation conformed to the Guide for the Care and Use of Laboratory Animals, published by the US National Institutes of Health (NIH).

Dimethyl Fumarate (Sigma, Aldrich) was administered via daily i.p. injection at a dose of 90 mg/kg. DMF was dissolved in DMSO (30 mg/ml) and mixed with pre-warmed (37 °C) vehicle (10% (2-Hydroxypropyl)-β-cyclodextrin (Sigma Aldrich) in PBS) immediately prior to injection. The volume of a single injection was 500 μl. SU5416 (Sigma Aldrich) was used as previously described^18^. administered subcutaneously once a week in a dose of 20 mg/kg. Bleomycin (Hospira) was delivered in Alzet osmotic miniature pumps (model 1007D) at a dose of 1.8 unit/mouse. For hypoxic conditions mice were placed in a hypoxic chamber (10% oxygen) (BioSpherix). Right ventricular pressures were measured with a high-fidelity pressure catheter 1.2 F Mikro-tip^®^ (Model SPR-1000, Millar Instruments) inserted in the right ventricle via cannulation of the right jugular vein. Pressure waveforms were monitored in real time with the PowerLab Chart 8 data acquisition system (ADInstruments) and ten minutes of stable pressure recordings were acquired for each mouse. RV pressures were calculated by averaging at least 60 cardiac cycles for each mouse. Transthoracic echocardiography was performed using a Vevo 770 High-Resolution Imaging System with 30-MHz RMV-707b scanning head (VisualSonics). Pulmonary acceleration time (PAT) and PAT as a fraction of ejection time (ET) were measured from the pulse-wave Doppler recordings of the PA blood flow as previously described^17^. For assessment of right ventricular hypertrophy the ventricles were excised and weighed. The left lung was fixed by tracheal perfusion either with 4% paraformaldehyde or 50% OCT (Thermo Fisher Scientific) in PBS and right lung used for RNA isolation.

For immunohistochemistry and immunofluorescent staining slides were processed and stained as described before^51^. Primary antibodies used in immunohistochemistry: HO-1 (Enzo), NOX4 (Abcam), vWF (Dako), CD45 (eBioscience), MAC3 (BD Biosciences), αSMA (Lab Vision/NeoMarkers), nitrotyrosine (EMD Millipore) and active β-catenin antibody (clone 8E7, Millipore).

For the quantification of immune cells, ImageJ (NIH, Bethesda, MD) was used to count DAPI positive nuclei (total number of cells) and MAC3 or CD45 positive cells from 5 fields of view per sample. For the nitrotyrosine staining the pictures were thresholded and average signal intensity quantified in ImageJ (5 fields of view per sample) For the HO-1 and NOX4 staining the immunoreactive vessels (CD31 or vWF positive) were quantified from 20 fields of view per sample. Quantification of pulmonary arteries muscularization was described before^17^.

### Cell Culture

Human pulmonary artery endothelial cells (HPAECs) were purchased from Lonza. IMR90 fetal lung fibroblasts were purchased from ATCC. Adult lung fibroblasts were obtained from patients with SSc who underwent lung transplantation or healthy control subjects and lung vascular smooth muscle cells from PH patients who underwent lung transplantation or healthy control subjects at the University of Pittsburgh Medical Center. Use of human tissues was approved by IRB or CORID (970946, PRO14010265 and CORID No. 300) at the University of Pittsburgh Medical Center. All experimental protocols were approved and carried out in accordance with the IRB and CORID guidelines. Written informed consent was obtained from all donors.

For TGFβ (PeproTech) treatment cells were pre-incubated in serum-free media for 16 h.

Cells were transfected with siRNA specific to human *NRF2, βTRCP* or scrambled siRNA (ON-TARGETplus SMART pool) using Lipofectamine RNAiMAX Transfection Reagent (Thermo Fisher Scientific) according to the manufacturer’s protocol.

Chromatin immunoprecipitation (ChIP) was performed as previously described^52^ using ChIP grade SP1 antibody (EMD Millipore). The amount of immunoprecipitated DNA (percent recovery) was calculated from a standard curve generated with the serial dilutions of input chromatin. A luciferase reporter driven by −353 to + 58 fragment (representing the wildtype Sp1 binding sites) of the human COL1A2 promoter was described previously^53^. For the NFκB activity reporter we used Ready-To-Glow™ NFkappaB Secreted Luciferase Reporter System (Clontech).

### RNA isolation, quantitative RT-PCR

Total RNA was isolated using TRIzol reagent (MRC, Inc.) for cell culture samples or by using the RNeasy Mini Kit (Qiagen) for tissue samples. Real-time PCR was performed using the StepOnePlus™ Real-Time PCR System using SYBR^®^ Green PCR Master Mix (Applied Biosystems). The primer sequences used are available upon request.

### Western blot

Cells were lysed and processed as described before^54^. Antibodies used: pSMAD2/3, SMAD2/3, pSMAD1/5, SMAD1/5, pSTAT3, STAT3, pcJUN, and cJUN from Cell Signaling, Lamin A/C, Sp1, and CTGF from Santa Cruz Biotechnology, Col I from Southern Biotech, β-catenin from Abcam, and HO-1 from Enzo.

### Statistical analysis

Prism 5 (GraphPad) was used for all analyses. Values are presented as means ± SD. Two-sided student t test was used for comparing 2 groups, α = 0.05. For analysis of more then two groups one-way ANOVA with a post hoc Tukey test was used, α = 0.05.

## Additional Information

**How to cite this article**: Grzegorzewska, A. P. *et al*. Dimethyl Fumarate ameliorates pulmonary arterial hypertension and lung fibrosis by targeting multiple pathways. *Sci. Rep.* **7**, 41605; doi: 10.1038/srep41605 (2017).

**Publisher's note:** Springer Nature remains neutral with regard to jurisdictional claims in published maps and institutional affiliations.

## Supplementary Material

Supplementary Figures
