# Supplementary material for: Dimethyl Fumarate ameliorates pulmonary arterial hypertension and lung fibrosis by targeting multiple pathways
Source: Sci Rep. 2017 Feb 2;7:41605. doi: 10.1038/srep41605 (PMC5288696; doi:10.1038/srep41605)
Supplement: Supplementary Figures [file srep41605-s1.pdf]

Supplementary Material

Dimethyl Fumarate ameliorates pulmonary arterial hypertension and lung fibrosis by targeting multiple pathways

Agnieszka P. Grzegorzewska[1], PhD, Francesca Seta[2], PhD, Rong Han[1], PhD, Caitlin A. Czajka[5], PhD, Katsunari Makino[1], MD, Lukasz Stawski[1], MS, Jeffrey S. Isenberg[5, 6], MD, MPH, Jeffrey L. Browning[4], PhD, Maria Trojanowska[1*], PhD

[1] Boston University School of Medicine, Arthritis Center/Rheumatology, Boston, MA
Current address: University of California San Francisco, Department of Medicine, San Francisco, CA

[2] Boston University School of Medicine, Vascular Biology Section, Boston, MA

[4] Boston University School of Medicine, Microbiology, Boston, MA

[5]Heart, Lung, Blood and Vascular Medicine Institute, University of Pittsburgh

[6]Division of Pulmonary, Allergy and Critical Care Medicine, University of Pittsburgh School of Medicine

*To whom correspondence should be addressed: trojanme@bu.edu

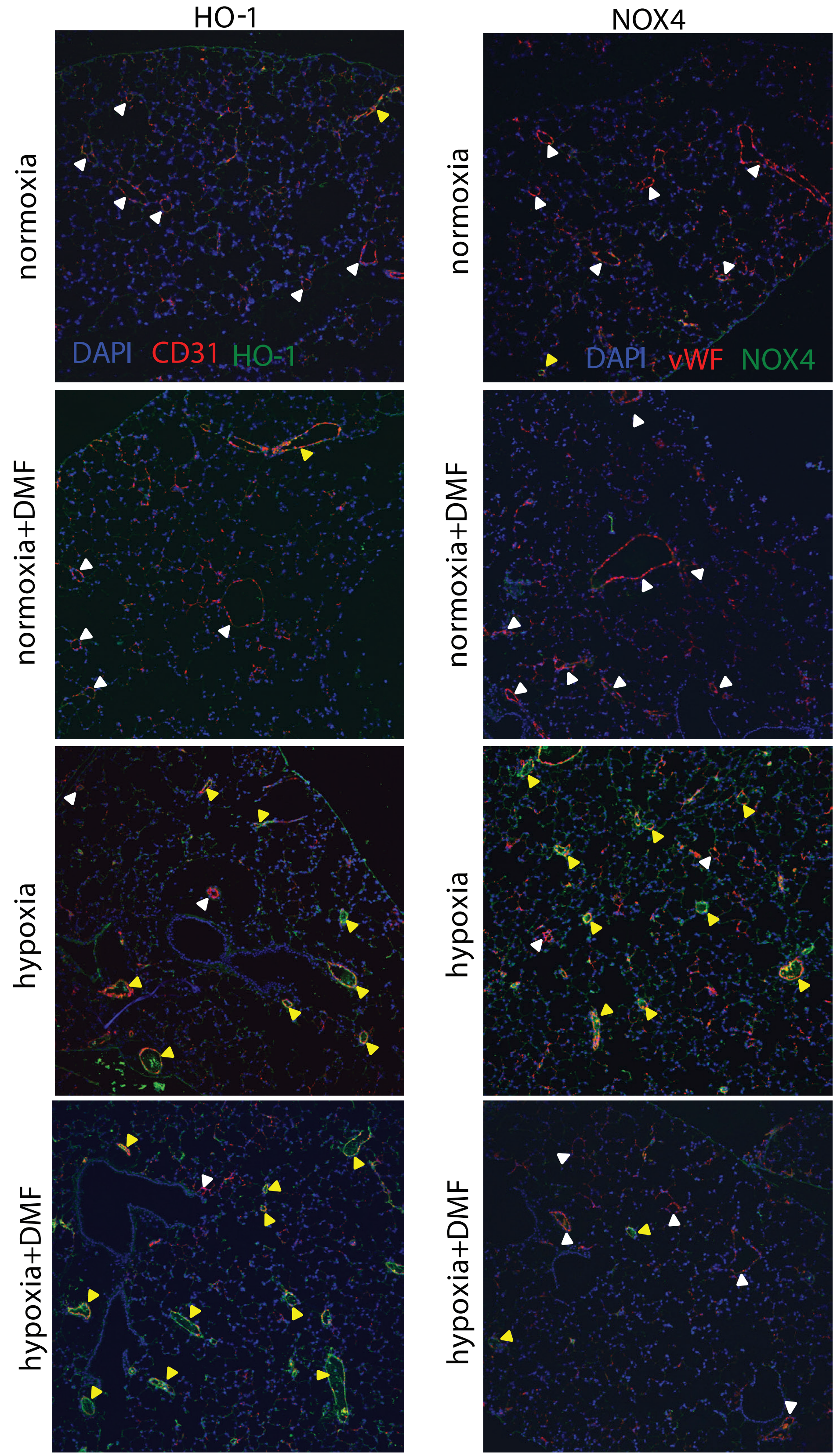

Supplementary Fig.1. DMF upregulates Ho-1 and reduced hypoxia-induced Nox4 in pulmonary vasculature

Representative pictures of double immunostained frozen lung sections of hypoxic mice from preventive treatment group. Sections were stained with CD31 or vWF as endothelial cells marker and HO-1 or NOX4 antibodies. White arrowheads indicate HO-1/NOX4 non-immunoreactive- and yellow arrowheads immunoreactive-vessels.

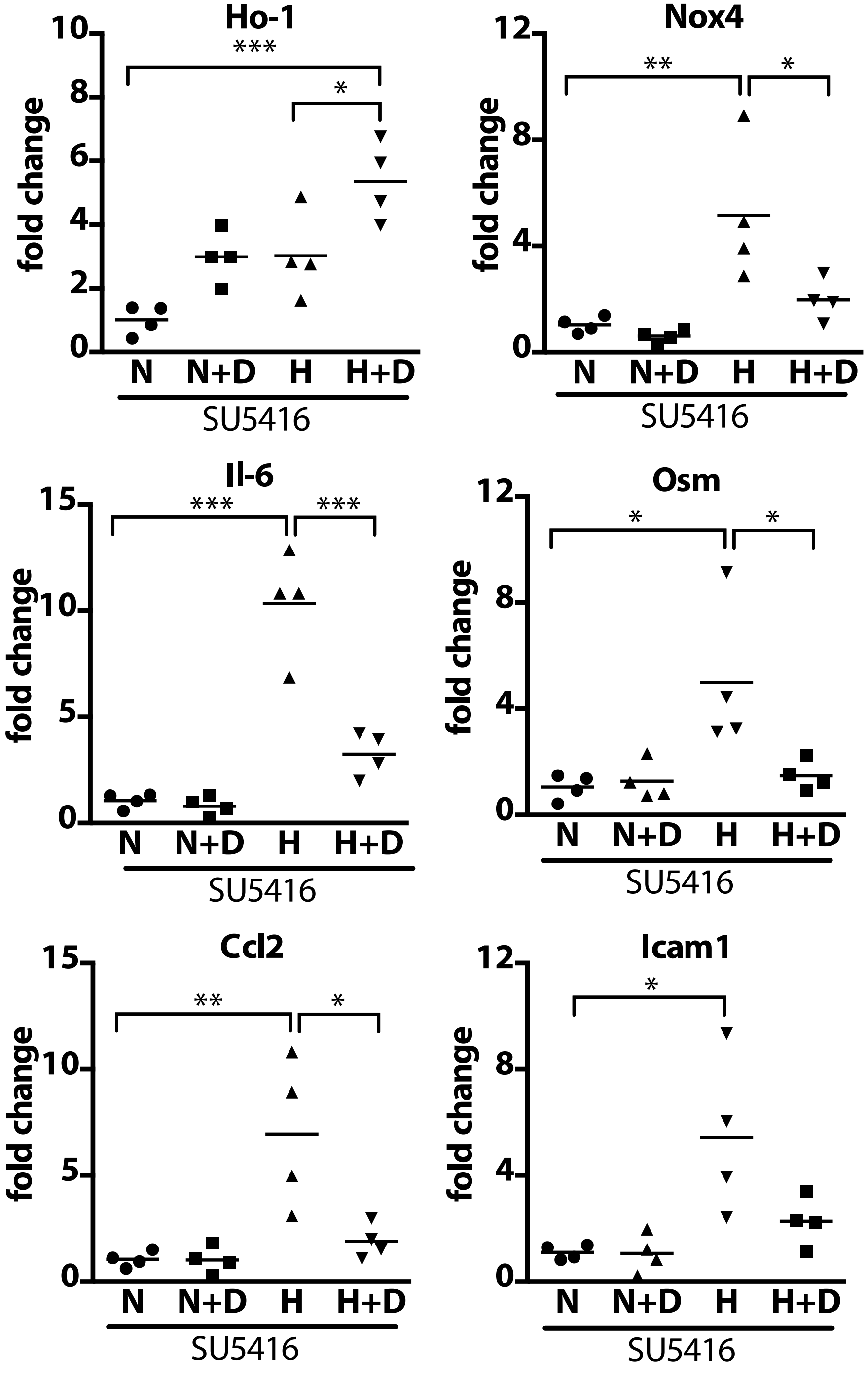

Supplementary Fig.2. DMF inhibits hypoxia/SU5416-induced expression of the pro-inflammatory genes in lung

qPCR quantification of relative mRNA levels in lung of chronic hypoxia/SU5416 mice. Normoxia (N), Hypoxia (H), DMF treatment (D). Normoxia (N), Hypoxia (H), DMF treatment (D). P values were calculated using the two-way ANOVA and Tukey's multiple comparisons test. *P<0.05, **P<0.01, ***P<0.001

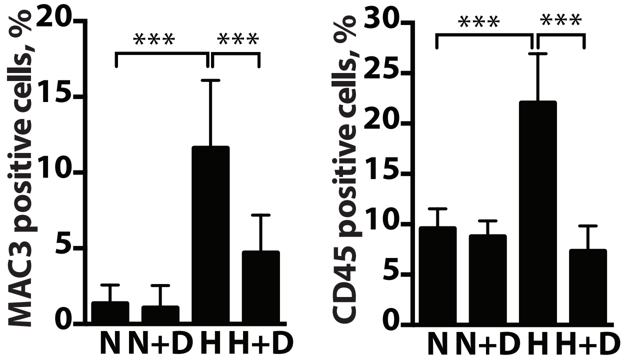

Supplementary Fig.3. DMF prevents hypoxia-induced immune cells tissue infiltration.

Frozen lung sections from the preventive mode experiment were immunostained using anti-MAC3 (**D**) antibody as a general macrophage marker and anti-CD45 (**E**) antibody as a general leukocyte marker. Positive cells were counted from 5 fields of view per mouse. n=4 mice per group, data shown as mean ± SD. *P<0.05, **P<0.01, ***P<0.001.

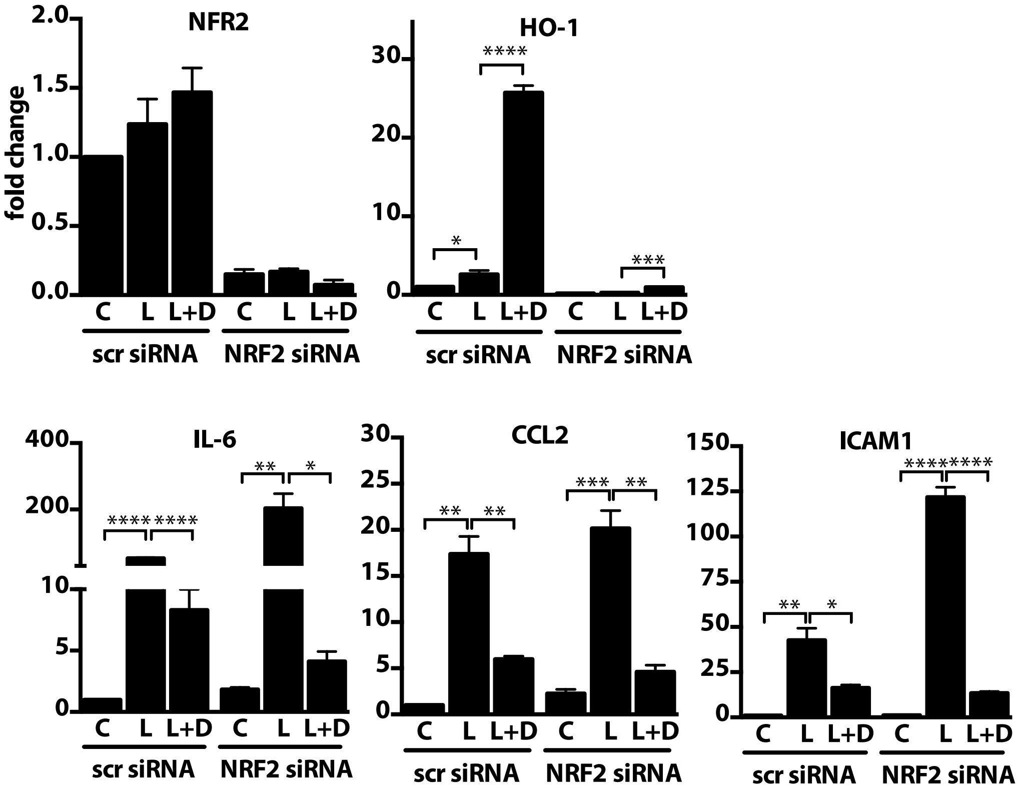

Supplementary Fig.4. NRF2 is not necessary for anti-inflammatory action of DMF in LPS-stimulated HPAECs

Human pulmonary arterial endothelial cells (HPAECs) were subjected to siRNA silencing with scrambled siRNA (scr) or NRF2 targeting siRNA for 48h and then treated with LPS (L) and 10 μM DMF (D) for 4h. Relative gene expression was measured with qPCR. N= 3 independent experiments. Data shown as mean ± SD. P values were calculated using the two-way ANOVA and Tukey's multiple comparisons test. *P<0.05, **P<0.01, ***P<0.001

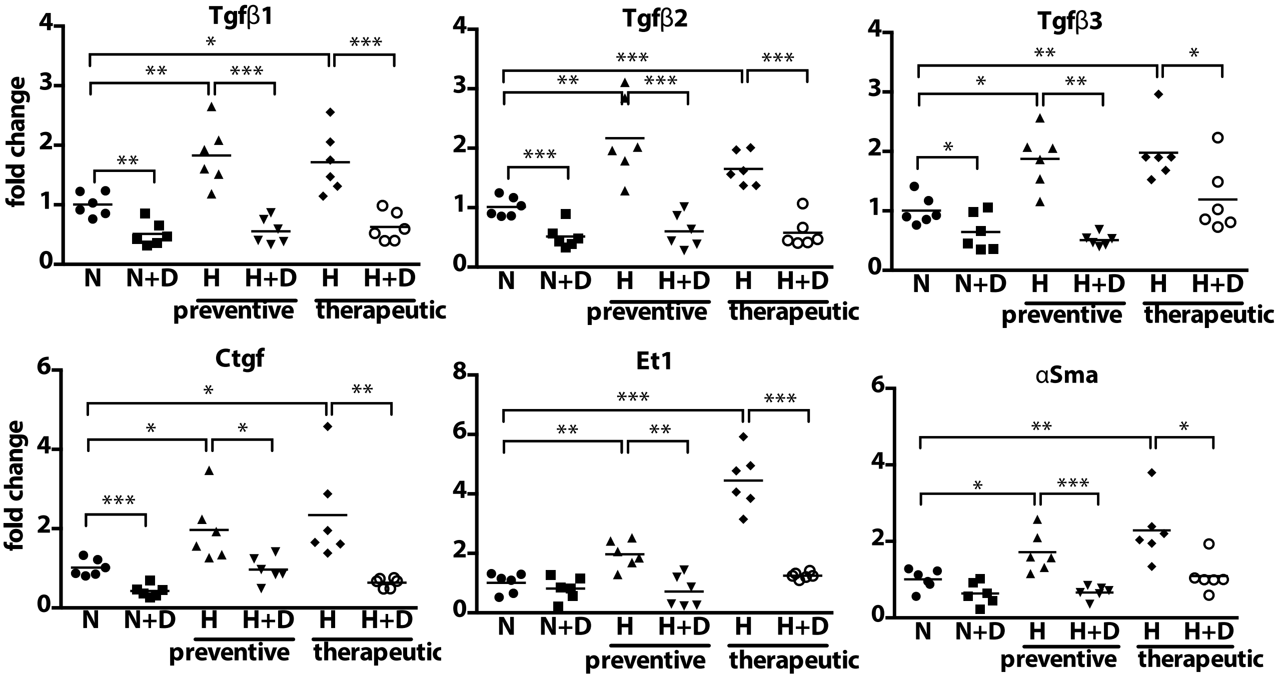

Supplementary Fig.5. DMF inhibits hypoxia-induced expression of the pro-fibrotic genes in lung

qPCR quantification of relative mRNA levels in lung of chronic hypoxia mice. Normoxia (N), Hypoxia (H), DMF treatment (D). Normoxia (N), Hypoxia (H), DMF treatment (D). P values were calculated using the two-way ANOVA and Tukey's multiple comparisons test. *P<0.05, **P<0.01, ***P<0.001

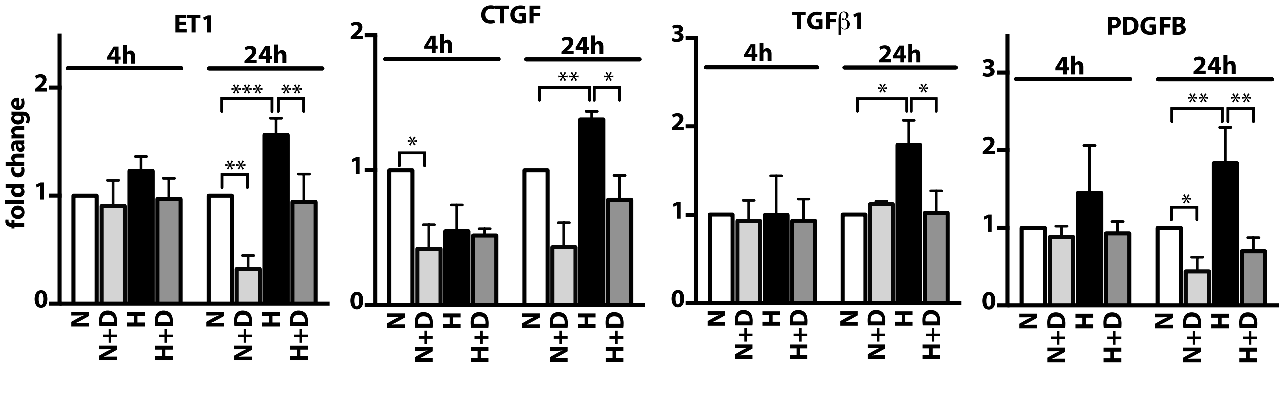

Supplementary Fig.6. DMF inhibits hypoxia-induced expression of pro-fibrotic and proliferative genes in HPAECs

HPAECs were incubated for up to 24h in (H) hypoxia (2.5% O_2) or (N) normoxia (21% O_2) with 10μM DMF (D) or DMSO. Relative gene expression was measured with qPCR. n=3 independent experiments. P values were calculated using the two-way ANOVA and Tukey's multiple comparisons test. Data shown as mean ± SD. *P<0.05, **P<0.01, ***P<0.001.

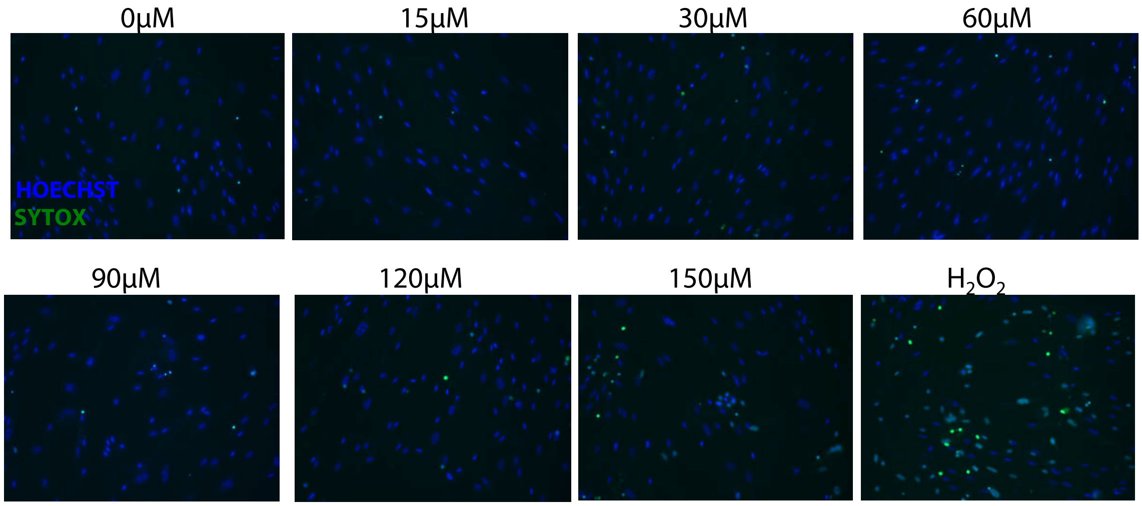

Supplementary Fig.7. DMF does not induce cytotoxicity in a range of concentration of 0- 120 μM.

IMR90 fibroblasts were treated with 0-120 μM DMF for 24h or H_2O_2 for 1h as a positive control of cell death. Cells were then stained with Hoechst for nuclei and SYTOX for dead cells.

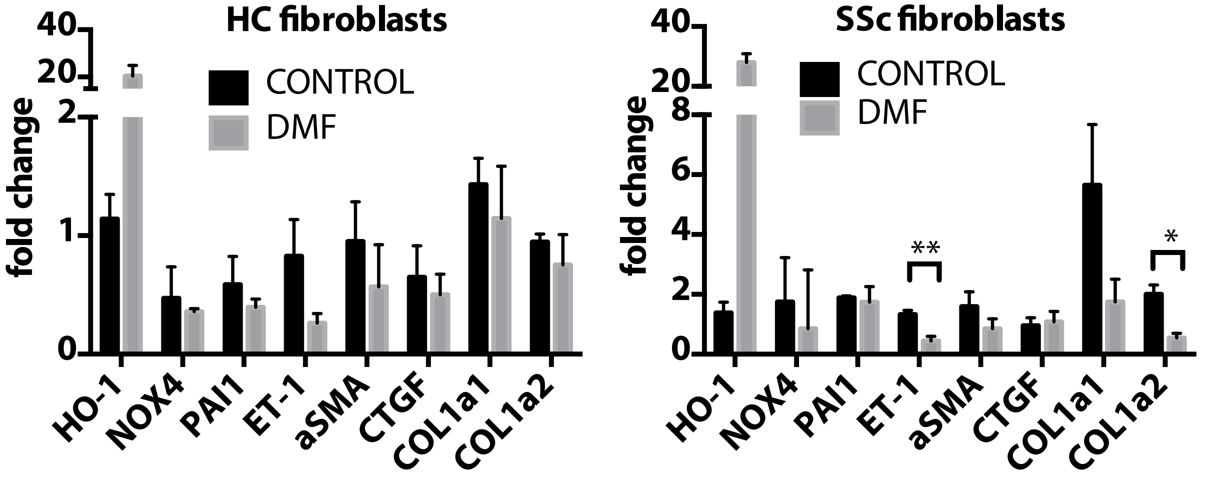

Supplementary Fig.8. Effect of DMF on basal levels of gene expression in lung fibroblasts

Human primary lung fibroblasts from healthy controls and scleroderma patients were treated for 24h with 90μM DMF and control cells were treated with vehicle (DMSO). Relative gene expression was measured with qPCR. . Data shown as mean ± SD. P values were calculated using the two-way ANOVA and Tukey's multiple comparisons test. *P<0.05, **P<0.01, ***P<0.001.

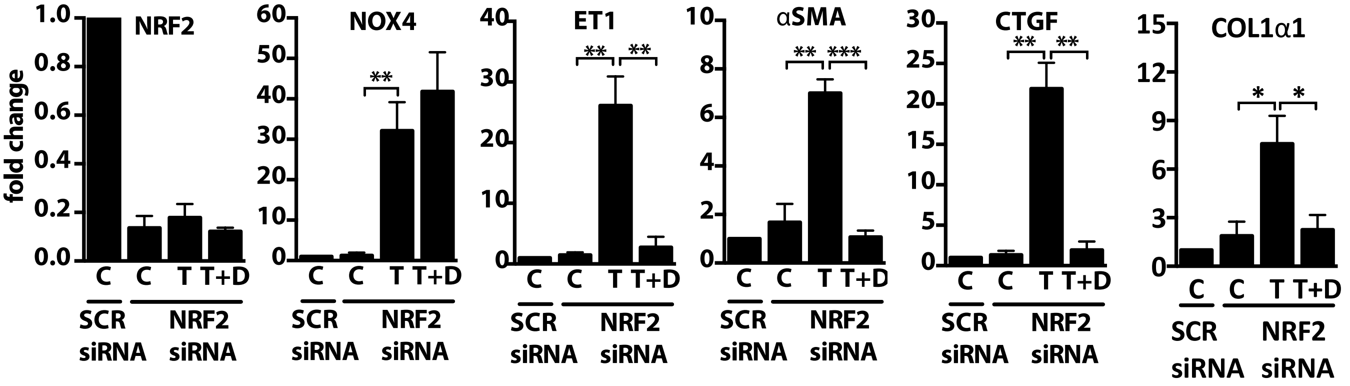

Supplementary Fig.9. NRF2 is not necessary for the anti-fibrotic action of DMF in lung fibroblasts treated with TGFβ

IMR90 cells were subjected to siRNA silencing with scrambled siRNA (scr) or NRF2 targeting siRNA for 48h and then treated with TGFβ (T) and 90 μM DMF (D) for 24h. Relative gene expression was measured with qPCR. N= 3 independent experiments. Data shown as mean ± SD. P values were calculated using the two-way ANOVA and Tukey's multiple comparisons test. *P<0.05, **P<0.01, ***P<0.001

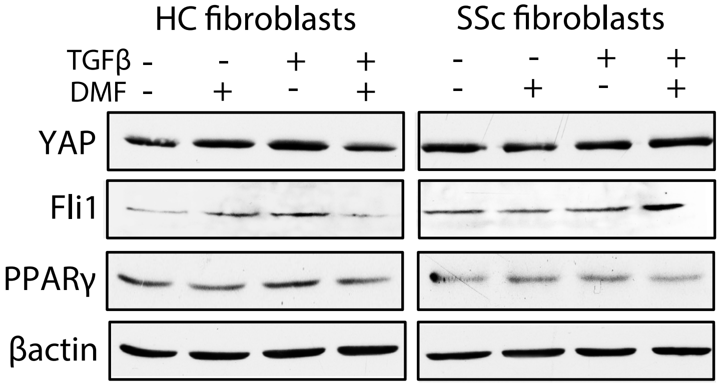

Supplementary Fig.10. DMF and TGFβ do not alter protein levels of YAP, Fli1 and PPARγ, nor the mRNA levels of TAZ and β-catenin

Human primary lung fibroblasts from healthy controls (HC) and scleroderma patients (SSc) were treated for 24h with 2.5ηg/ml TGFβ and 90μM DMF, control cells (C) were treated with vehicle (DMSO). Representative pictures of blots are shown.

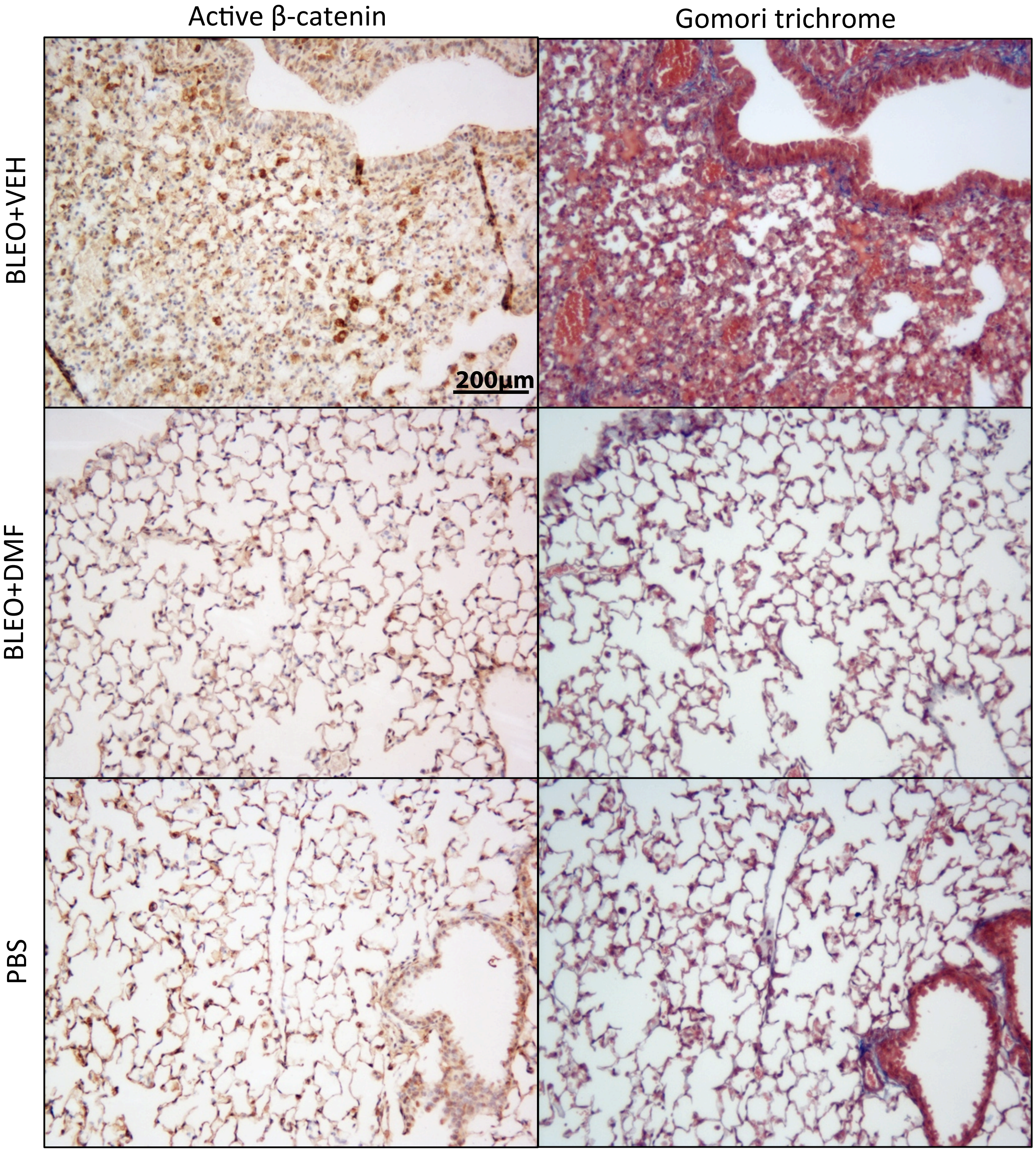

Supplementary Fig.11. Lung fibrotic regions are enriched in active β-catenin positive cells

Parrafin embedded lung sections of bleomycin (BLEO) and DMF treated mice were stained with an antibody against active (unphosphorylated) β-catenin or Gomori trichrome for collagen.

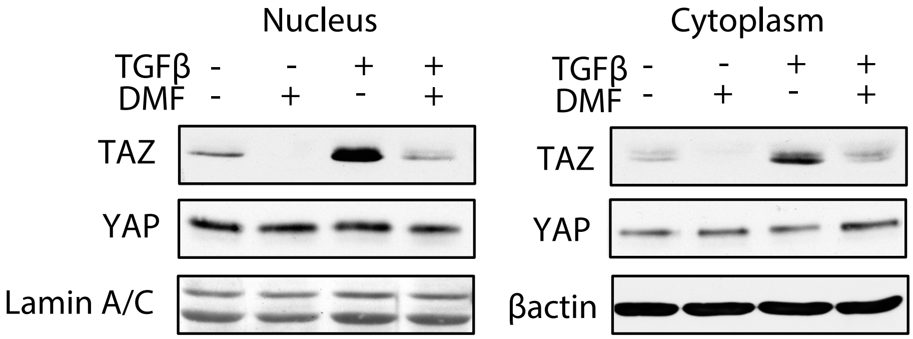

Supplementary Fig.12. DMF does not alter nuclear-cytoplasmic shuttling of YAP/TAZ
IMR90 cells were treated for 1h with 90μM DMF and then for 1h with 2.5ηg/ml TGFβ, control cells were treated with vehicle (DMSO). Cells were collected and subjected to cytoplasmic/ nuclear protein fractionation. Representative pictures of blots are shown.

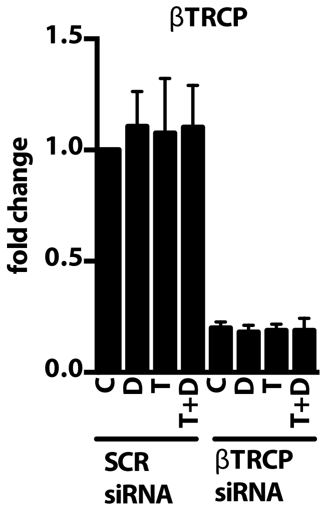

Supplementary Fig.13. Silencing efficiency of βTRCP with siRNA in lung fibroblasts
IMR90 cells were subjected to siRNA silencing with scrambled siRNA (scr) or βTRCP targeting siRNA for 48h and then treated with TGFβ (T) and 90 μM DMF (D) for 24h. Relative gene expression was measured with qPCR. N= 3 independent experiments. Data shown as mean ± SD.

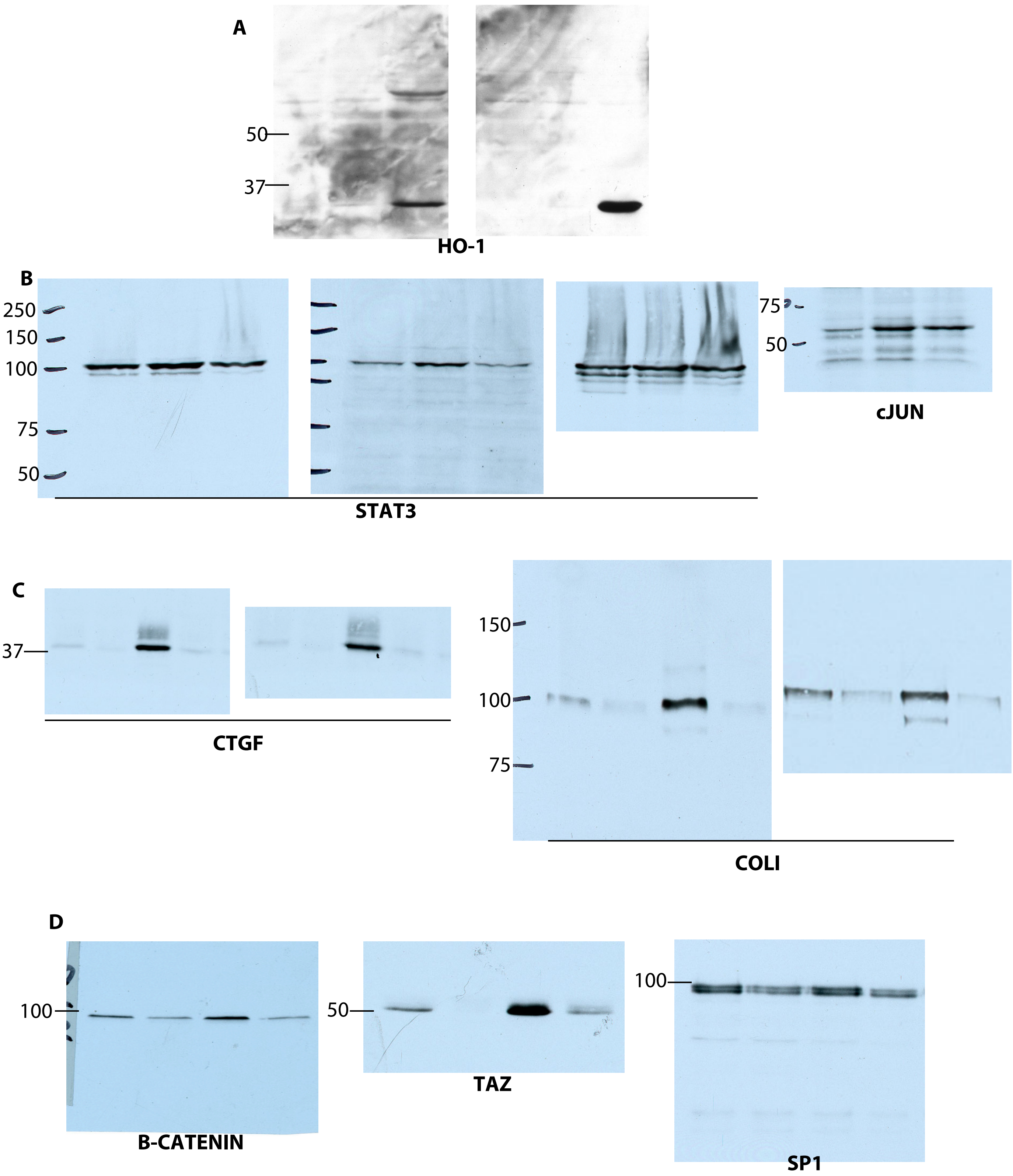

Supplementary Fig.14. Un-cropped images of immunoblots
(A) Immunoblots from Fig.3A. **(B)** Immunoblots from Fig.3D. **(C)** Immunoblats from Fig.6A. **(D)** Immunoblots from Fig.7A,D.
